# Transcriptome-wide association study reveals novel susceptibility genes for coronary atherosclerosis

**DOI:** 10.3389/fcvm.2023.1149113

**Published:** 2023-06-07

**Authors:** Qiuping Zhao, Rongmei Liu, Hui Chen, Xiaomo Yang, Jiajia Dong, Minfu Bai, Yao Lu, Yiming Leng

**Affiliations:** ^1^ Heart Center of Henan Provincial People’s Hospital, Fuwai Central China Cardiovascular Hospital, Zhengzhou, China; ^2^ School of Life Course Sciences, King’s College London, London, United Kingdom; ^3^ Clinical Research Center, The Third Xiangya Hospital, Central South University, Changsha, China; ^4^ Department of Cardiology, The Third Xiangya Hospital, Central South University, Changsha, China

**Keywords:** GWAS, coronary atherosclerosis, TWAS, genetic risk factors, genetic mechanisms

## Abstract

**Background:**

Genetic risk factors substantially contributed to the development of coronary atherosclerosis. Genome-wide association study (GWAS) has identified many risk loci for coronary atherosclerosis, but the translation of these loci into therapeutic targets is limited for their location in non-coding regions. Here, we aimed to screen the potential coronary atherosclerosis pathogenic genes expressed though TWAS (transcriptome wide association study) and explore the underlying mechanism association.

**Methods:**

Four TWAS approaches (PrediXcan, JTI, UTMOST, and FUSION) were used to screen genes associated with coronary atherosclerosis. Enrichment analysis of TWAS-identified genes was applied through the Metascape website. The summary-data-based Mendelian randomization (SMR) analysis was conducted to provide the evidence of causal relationship between the candidate genes and coronary atherosclerosis. At last, the cell type-specific expression of the intersection genes was examined by using human coronary artery single-cell RNA-seq, interrogating the immune microenvironment of human coronary atherosclerotic plaque at different stages of maturity.

**Results:**

We identified 19 genes by at least three approaches and 1 gene (*NBEAL1*) by four approaches. Enrichment analysis enriching the genes identified at least by two TWAS approaches, suggesting that these genes were markedly enriched in asthma and leukocyte mediated immunity reaction. Further, the summary-data-based Mendelian randomization (SMR) analysis provided the evidence of causal relationship between *NBEAL1* gene and coronary atherosclerosis, confirming the protecting effects of *NBEAL1* gene and coronary atherosclerosis. At last, the single cell cluster analysis demonstrated that *NBEAL1* gene has differential expressions in macrophages, plasma cells and endothelial cells.

**Conclusion:**

Our study identified the novel genes associated with coronary atherosclerosis and suggested the potential biological function for these genes, providing insightful guidance for further biological investigation and therapeutic approaches development in atherosclerosis-related diseases.

## Introduction

Coronary artery disease (CAD), a leading global cause of death, is influenced by lifestyle, interactions of environmental, genetic risk factors and so on (1). Environmental and lifestyle factors were well-established coronary atherosclerosis risk factors, including physical activity, body mass index (BMI), smoking, healthy diet score and blood pressure (BP), total cholesterol (TC) and fasting plasma glucose (FPG), as defined by American Heart Association (2, 3).

Meanwhile, genetic risk factors substantially contributed to the development of coronary atherosclerosis (4, 5). A study of more than 20,000 Swedish twins confirmed a heritability of ∼50% for fatal coronary atherosclerosis among close relatives. Another analysis study using updated genome-wide approaches similarly quantified the heritability of coronary atherosclerosis at 40%–50% (6, 7). Recently, the genome-wide approaches have laid the foundation to understand the underlying genetic architecture of coronary atherosclerosis, to uncover novel biology and to apply these findings to clinical practice. More than 250 risk loci for coronary atherosclerosis have been identified though genome-wide association study (GWAS), helping to inform experimental interrogation of putative causal mechanisms for coronary atherosclerosis (8).

However, the translation of these loci into therapeutic targets is limited. One of the possible reasons is that most of these risk loci are located in the non-coding region of the human genome. The biological explanations are thus not straightforward. In order to solve this problem, TWAS (transcriptome wide association study) has been developed to identify and prioritize disease genes. TWAS may point to causal genes at risk sites identified by GWAS, thereby providing further insight into biological mechanisms (9, 10). In addition, TWAS can provide higher sensitivity to identify susceptibility genes missed by traditional GWAS analyses.

In this study, four different TWAS methods were used to systematically prioritize the potential coronary atherosclerosis pathogenic genes expressed in coronary arteries tissues, and to further reveal the underlying mechanism association through pathway enrichment analysis, providing novel evidence for the genetic mechanisms of coronary atherosclerosis.

## Methods

### Study design

First, we extracted the complete summary data from the GWASs for coronary atherosclerosis. Then we performed a TWAS analysis using four different methods with pre-trained gene expression models. Third, the summary-data-based Mendelian randomization (SMR) was used to assess the causal relationship between the intersection of genes and coronary atherosclerosis risk. Finally, we utilized public single-cell transcriptome data to explore the cell type-specific expression of the intersection genes in the coronary artery.

### The data source for gene-expression models and coronary atherosclerosis

We used the recently released data of the Genotype-Tissue Expression (GTEx, https://gtexportal.org/home/) project (V8), which includes RNA sequencing data and whole-genome sequencing (WGS) data of coronary artery (*N* = 213). The training methods of gene-expression models can be found in previous studies (11–13). We utilized the pre-trained prediction models from Zenodo (https://doi.org/10.5281/zenodo.3842289) and TWAS/FUSION website (https://s3.us-west-1.amazonaws.com/gtex.v8.fusion/EUR/GTExv8.EUR.Artery_Coronary.tar.gz) for further transcriptome-wide association analyses. We collected the GWAS summary data of coronary atherosclerosis from FinnGen, a significant public-private partnership that aims to gather and analyze genetic and health data from more than 500,000 people. The latest release is from December 2022, including 342,499 participants (190,879 females and 151,620 males) and 20,175,454 variants. The diagnosis of coronary atherosclerosis from the hospital discharge registry and cause of death registry was based on the International Classification of Disease. In total, 42,421 cases and 285,621 controls were identified (https://www.finngen.fi/en/access_results).

### Transcriptome-wide association study

We performed a summary-based TWAS using four different approaches, including the joint-tissue imputation (JTI) method (11), the PrediXcan (12), the modified unified test for molecular signatures (UTMOST) (11, 13), and the FUSION (14). Overall, the JTI borrows information on each tissue-tissue pair (or cell type) to improve the prediction quality. The PrediXcan uses the elastic net to determine the optimal hyperparameter. UTMOST borrows information across tissues using a sparse group-LASSO method. Similarly, FUSION is a suite of tools for performing TWAS. A combination of complementary methods may improve the reliability of results. We also applied Bonferroni corrections for multiple comparisons, considering the total number of tested genes across different methods.

### Summary-data-based Mendelian randomization

We further validated the TWAS results using the SMR analysis followed by the heterogeneity in dependent instrument (HEIDI) test (15). The SMR was used to test for the potential causal effect of the expression level of a gene on coronary atherosclerosis using summary GWAS data and expression quantitative trait loci (eQTLs) studies. Genes were considered plausible causal gene only if they passed both SMR and HEIDI tests (*P*_SMR_ < 0.05 and HEIDI *P* > 0.05).

### Enrichment analysis and cell-type specificity analysis

We performed the enrichment analysis of TWAS-identified genes using the Metascape website (16). We examined the cell type-specific expression of the intersection genes by using human coronary artery single-cell RNA-seq, interrogating the immune microenvironment of human coronary atherosclerotic plaque at different stages of maturity. Clusters were annotated by taking default parameters with an online tool to visualize single-cell data (17). Finally, we performed differential expression analysis to determine whether these candidate genes were differentially expressed in some specific cell types.

## Results

### Transcriptome-wide significant genes for coronary atherosclerosis

**Figure 1:**
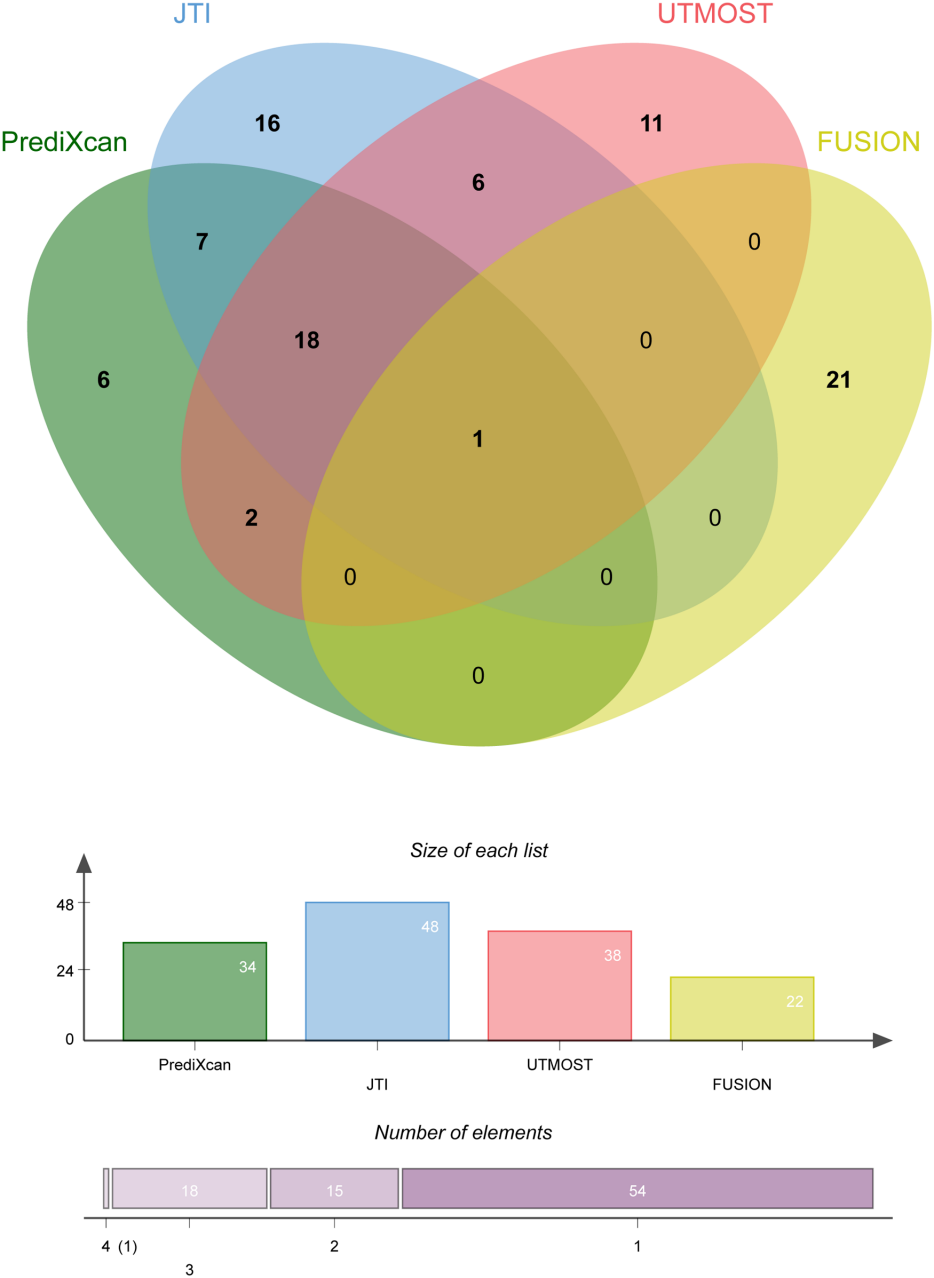
Overlap analysis of the coronary atherosclerosis associated genes by different TWAS approaches.

Four TWAS approaches (PrediXcan, JTI, UTMOST, and FUSION) were used to screen genes associated with coronary atherosclerosis (Supplementary Tables S1–S4). Finally, we identified 19 genes with TWAS *P-*value passing multiple testing, such as *NBEAL1*, *CEACAM19*, *AC243964.3*, *INO80E*, et al. Among these candidate genes, only one gene (*NBEAL1*) was identified by all TWAS approaches. Figure 1 shows the Venn diagram of the TWAS-identified genes. Table 1 summarizes the 19 associated genes identified by at least three TWAS approaches.

**Table 1:** TWAS-identified genes associated with coronary atherosclerosis.

	PrediXcan		JTI		UTMOST		FUSION	
	*Z* score	*P* value	*Z* score	*P* value	*Z* score	*P* value	*Z* score	*P* value
NBEAL1	−5.16	2.47 × 10^−7^	−5.26	1.43 × 10^−7^	−5.02	5.12 × 10^−7^	−7.62	2.43 × 10^−14^
CEACAM19	11.85	2.13 × 10^−32^	9.12	6.90 × 10^−20^	9.43	3.88 × 10^−21^	–	–
AC243964.3	9.37	6.98 × 10^−21^	7.82	4.93 × 10^−15^	7.35	1.95 × 10^−13^	–	–
INO80E	6.50	8.30 × 10^−11^	6.26	3.74 × 10^−10^	6.59	4.33 × 10^−11^	–	–
WNT3	−6.09	1.10 × 10^−9^	−5.86	4.51 × 10^−9^	−6.13	8.52 × 10^−10^	–	–
C17orf107	6.09	1.15 × 10^−9^	5.01	5.28 × 10^−7^	5.18	2.13 × 10^−7^	–	–
SCIMP	6.06	1.34 × 10^−9^	6.04	1.48 × 10^−9^	4.84	1.25 × 10^−6^	–	–
HLA-DQA2	−5.81	6.09 × 10^−9^	−5.87	4.29 × 10^−9^	−5.47	4.36 × 10^−8^	–	–
YPEL3	−5.81	6.25 × 10^−9^	−4.92	8.58 × 10^−7^	−6.05	1.40 × 10^−9^	–	–
KANSL1-AS1	−5.71	1.08 × 10^−8^	−5.61	1.99 × 10^−8^	−5.36	7.98 × 10^−8^	–	–
LACTB	5.48	4.05 × 10^−8^	5.65	1.55 × 10^−8^	5.34	9.07 × 10^−8^	–	–
SLC26A1	−5.45	4.89 × 10^−8^	−5.10	3.31 × 10^−7^	−5.00	5.65 × 10^−7^	–	–
KAT8	−5.39	6.95 × 10^−8^	−6.27	3.50 × 10^−10^	−6.08	1.18 × 10^−9^	–	–
EPHX2	5.38	7.27 × 10^−8^	5.10	3.28 × 10^−7^	4.86	1.16 × 10^−6^	–	–
CHRNE	5.26	1.41 × 10^−7^	5.30	1.11 × 10^−7^	5.33	9.68 × 10^−8^	–	–
LRRC37A2	−5.25	1.51 × 10^−7^	−5.51	3.44 × 10^−8^	−5.19	2.10 × 10^−7^	–	–
NDUFAF6	4.78	1.68 × 10^−6^	5.00	5.73 × 10^−7^	5.43	5.61 × 10^−8^	–	–
AC135050.3	−4.65	3.29 × 10^−6^	−4.71	2.38 × 10^−6^	−4.50	6.55 × 10^−6^	–	–
EARS2	4.46	7.97 × 10^−6^	4.72	2.34 × 10^−6^	4.68	2.83 × 10^−6^	–	–

### Enrichment analysis of the TWAS-identified genes

**Figure 2:**
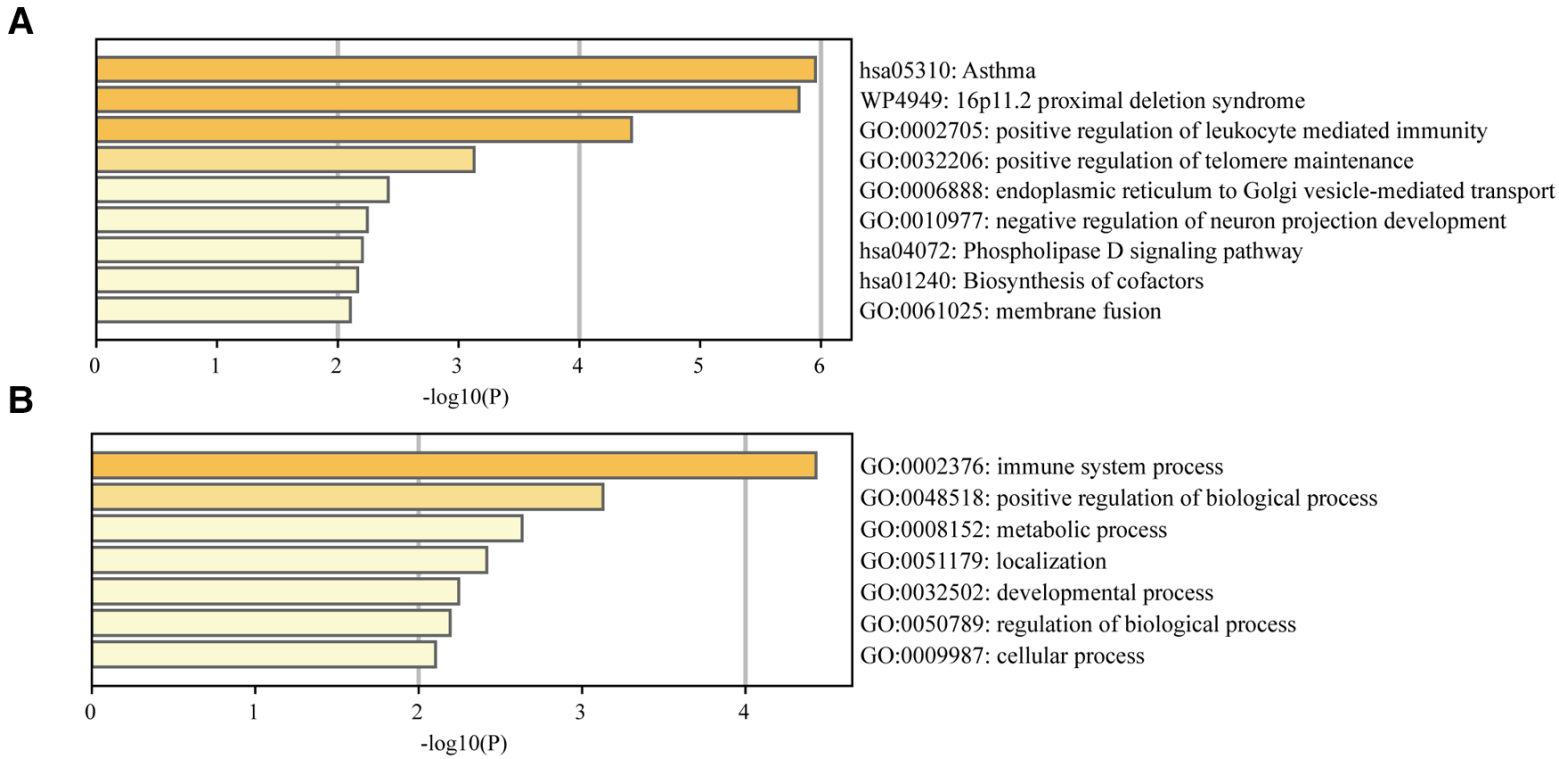
(**A**) Enrichment analysis of the TWAS-identified genes. (**B**) Enrichment analysis of the immune system process.

Enrichment analysis of the genes identified at least by two TWAS approaches results were shown in Figure 2. Several significant biological process terms and pathways were detected, such as asthma (hsa05310), positive regulation of telomere maintenance (GO:0032206), positive regulation of biological process (GO:0048518), negative regulation of neuron projection development (GO:0010977), immune system process (GO:0002376) and so on. We found that among all the significant biological process terms and pathways, the genes identified at least by two TWAS approaches were markedly enriched in asthma and leukocyte mediated immunity reaction (Figure 2).

### Causal relationships between *NBEAL1* and coronary atherosclerosis

*The NBEAL1* gene was identified by all the four TWAS approaches, suggesting that this gene might be the most reliable associated gene. We therefore explored the causal relationship between *NBEAL1* expression in the coronary artery and coronary atherosclerosis using SMR. Our results showed *NBEAL1* was a plausible causal gene in the coronary artery and coronary atherosclerosis. And it provided a protective role in coronary atherosclerosis incidence (odds ratio (OR) = 0.84, 95% confidence interval (CI) = 0.79–0.90, *P*-value = 5.84^−8^) (Supplementary Table S5), showing the same direction as that in TWAS analysis.

### Single-cell cluster analysis

**Figure 3:**
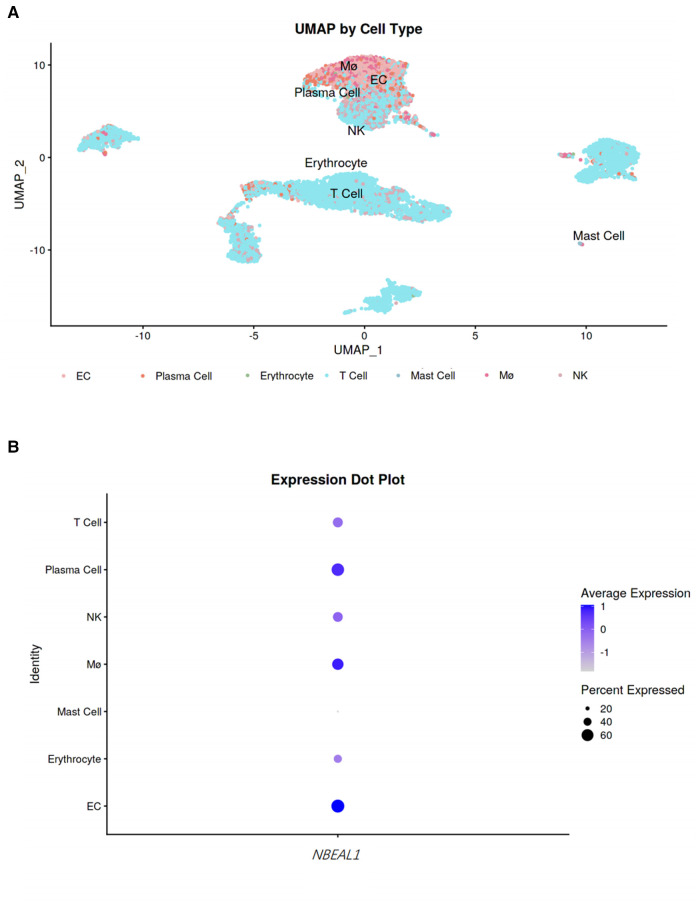
Single cell cluster analysis. (**A**) UMAP by cell type. To observe the expression of *NBEAL1* gene in cells, the darker the color, the higher the expression. (**B**) Expression dot plot. The size of the dot represents the proportion of the *NBEAL1* gene in the immune cells and the shade of the dot represents the degree of expression, the darker the color, the higher the expression.

To analyze the expression of *NBEAL1* gene in immune cells during the immune response process, we also performed single cell cluster analysis. Our results showed the human coronary atherosclerotic plaque tissue were annotated into seven clusters, including endothelial cells, plasma cells, erythrocyte, T cells, mast cells, macrophages, and Natural killer (NK) cells. The single cell cluster analysis demonstrated that *NBEAL1* gene has differential expressions in macrophages, plasma cells and endothelial cells (Figure 3).

## Discussion

In this study, we identified 19 genes by at least three TWAS approaches and one gene (*NBEAL1*) by four TWAS approaches. Next, we found that these genes were markedly enriched in asthma and leukocyte mediated immunity reaction though gene enrichment analysis approaches. Further, the SMR analysis provide the evidence of causal relationship between *NBEAL1* gene and coronary atherosclerosis, confirming the protective effects of *NBEAL1* gene and coronary atherosclerosis. Finally, the single cell cluster analysis demonstrated that *NBEAL1* gene has differential expressions in macrophages, plasma cells and endothelial cells.

CAD is one kind of cardiovascular diseases with a high prevalence rate, and its etiology is complex, among which genetic factors play the primary role. Although GWAS has identified many risk loci for CAD, only a minority of candidate genes could be experimentally demonstrated for their potential causal role in atherosclerosis. TWAS, a bioinformatics method based on expression levels of specific genes in defined tissues, could shed further insights into biological mechanisms in the pathophysiological process of diseases. There has been TWAS analysis identifying 18 novel genes in association with CAD based on two genetics-of-gene-expression panels (STARNET and GTEx) (18). More TWAS analyses are needed to explore genes associated with coronary atherosclerosis diseases.

Atherosclerosis is a chronic, complex inflammatory disease that is mediated by adaptive and innate immunity (19, 20). However, the specific molecular mechanisms and gene associated causal effects on coronary atherosclerosis are still unclear. Our study identified 19 novel genes by three TWAS approaches and one novel gene (*NBEAL1*) by four TWAS approaches associated with coronary atherosclerosis, and all these genes could be enriched in leukocyte mediated immunity reaction pathway. Abnormal immune response could interact with inflammation, metabolic risk factors, and other effector molecules to initiate and activate lesions in the arterial tree, inducing and accelerating the progression of coronary atherosclerosis (21, 22). Leukocytes, as the body's predominant immune cells, have traditionally been recognized as markers of acute or chronic inflammation. Previous studies have reported leukocytes and their subpopulations (lymphocytes, neutrophils, monocytes, eosinophils, and basophils) are associated with CAD (23, 24). Our recent research has also confirmed the causal relationship between leukocytes and CAD though Mendelian randomization (MR) approach. This study reconfirmed the relationship between leukocyte-mediated immune response and coronary atherosclerosis at the level of gene and gene function.

In addition, the study found the genes identified by TWAS could also be enriched in asthma pathway. There have been many researches focusing on the relationship between asthma and CAD. Some studies demonstrated that adult-onset asthma was associated with CAD, especially in females (25, 26), and the potential mechanism may involve systemic inflammation and cellular immunity. However, some bioinformatics analyses (such as MR) found that asthma was a causal factor for atrial fibrillation and heart failure, but not for CAD (27, 28). Therefore, more research is needed to explore the relationship between asthma and coronary atherosclerosis.

The *NBEAL1* is a new coronary atherosclerosis associated maker gene screened by four TWAS approaches in our study. The *NBEAL1* gene, located on human chromosome 2q33–2q34 was consisted of 25 exons spanning about 73 kb of the human genome. *NBEAL1* gene transcripts showed high expression in the human brain, kidney, prostate, and testis while low expression in the ovary, small intestine, colon and peripheral blood leukocyte. *NBEAL1* was first found higher expression in glioma tissues compared to the normal brain tissue, suggesting its correlation with the glioma (29). Besides, some research also revealed its association with stroke, cerebral small vessel disease and hereditary breast cancer (30–32).

Recently, a gene-based analyses from the NIH Exome Sequencing Project has identified the association between *NBEAL1* gene and early onset myocardial infarction, emphasizing the potential contributions of genetic variation in *NBEAL1* to the pathogenesis of premature atherosclerosis (33). However, there is few reports on the causal relationship between *NBEAL1* gene and coronary atherosclerosis. In this research, we further identified the causal relationship between *NBEAL1* gene and coronary atherosclerosis though SMR analysis, and we found *NBEAL1* gene was a protective causal gene maker for CAD. Combined with our previous enrichment analysis results, we speculate that *NBEAL1* gene might mediate the development of coronary atherosclerosis through the immune inflammatory pathway. Christian et al. found that *NBEAL1* is shown to be expressed most abundantly in arteries and could regulate cholesterol metabolism through modulation of LDLR expression in a mechanism which involves interaction with SCAP and PAQR3 and subsequent SREBP2-processing (34). Low expression of *NBEAL1* may lead to increased risk of CAD by downregulation of LDLR levels. In depth, it is still unclear whether *NBEAL1* gene associated immune inflammatory reaction could lead to the occurrence and development of coronary atherosclerosis though cholesterol metabolism pathway, and more research are needed to identify this.

Further, single cell cluster analysis found that *NBEAL1* gene has differential expressions in macrophages, plasma cells and endothelial cells. All these cells are the key cells involved in the occurrence and development of coronary atherosclerosis. Macrophages contribute to the maintenance of the local inflammatory response by producing reactive oxygen and nitrogen species and secreting chemokines, proinflammatory cytokines (including IL-6, TNF-α and IL-1β) (35–37). In pathological condition, the inflammatory cycle can be amplified with increased retention of lipoproteins (38, 39), finally promoting the formation of complicated atherosclerotic plaques. Endothelial cells are important barrier covering the wall of the arteries, regulating vascular tone, preventing platelet aggregation, and maintaining fluid homeostasis (40–42). Endothelial dysfunction plays a central role in all phases of the atherosclerotic process. Once more, the single cell cluster analysis results illustrate that the *NBEAL1* gene enriched in these key cells may be an important intervention target for prevention and treatment of coronary atherosclerosis.

However, there are still some limitations in our study. We did not exhibit molecular biology experiment to further explore the specific mechanism association between the maker genes and coronary atherosclerosis, and we will perform this part of research in future. Besides, our research results are obtained through bioinformatics methods, and we did not revalidate our findings in the coronary atherosclerosis population. This is also an important part of our future research directions.

## Conclusion

In conclusion, we identified the novel genes associated with coronary atherosclerosis and suggested the potential biological function (inflammatory immune pathway) for these genes, providing insightful guidance for further research and therapeutic approaches development in atherosclerosis-related diseases.

## Data Availability

The original contributions presented in the study are included in the article/**Supplementary Material**, further inquiries can be directed to the corresponding author.

## Supplementary material

SUPPLEMENTARY FIGURE S1

Transcriptome comparison of NBEAL1 gene in publicly available data (GSE43292). G1 is for control group, G2 is for experimental group.
