# Supplementary material for: Transcriptome-wide association study reveals novel susceptibility genes for coronary atherosclerosis
Source: Front Cardiovasc Med. 2023 Jun 7;10:1149113. doi: 10.3389/fcvm.2023.1149113 (PMC10282549; doi:10.3389/fcvm.2023.1149113)
Supplement: Supplementary file 1 [file Table1.docx]

Supplementary Table S1—S5

Table S1 TWAS-identified genes associated with coronary atherosclerosis (PrediXcan)

| gene | gene_name | zscore | effect_size | *P* value | var_g | pred_perf_r2 | pred_perf_pval | pred_perf_qval | n_snps_used | n_snps_in_cov | n_snps_in_model |
| --- | --- | --- | --- | --- | --- | --- | --- | --- | --- | --- | --- |
| ENSG00000186567 | CEACAM19 | 11.85069296 | 0.217316 | 2.13E-32 | 0.181301 | 0.226906 | 1.83E-13 | 4.99E-12 | 45 | 47 | 47 |
| ENSG00000266903 | AC243964.3 | 9.373987468 | 0.119281 | 6.98E-21 | 0.24174 | 0.311022 | 8.30E-19 | 3.95E-17 | 19 | 19 | 19 |
| ENSG00000169592 | INO80E | 6.495103157 | 0.140027 | 8.30E-11 | 0.07284 | 0.096604 | 3.76E-06 | 3.92E-05 | 10 | 10 | 10 |
| ENSG00000108379 | WNT3 | -6.093478012 | -0.10684 | 1.10E-09 | 0.110497 | 0.124249 | 1.26E-07 | 1.66E-06 | 20 | 22 | 22 |
| ENSG00000205710 | C17orf107 | 6.087467817 | 0.086745 | 1.15E-09 | 0.160357 | 0.185847 | 4.74E-11 | 9.71E-10 | 38 | 38 | 38 |
| ENSG00000161929 | SCIMP | 6.062270746 | 0.15682 | 1.34E-09 | 0.047733 | 0.092264 | 6.36E-06 | 6.32E-05 | 19 | 19 | 19 |
| ENSG00000237541 | HLA-DQA2 | -5.814284181 | -0.0552 | 6.09E-09 | 0.464009 | 0.653409 | 1.91E-50 | 3.26E-47 | 37 | 39 | 39 |
| ENSG00000090238 | YPEL3 | -5.810057616 | -0.09482 | 6.25E-09 | 0.125439 | 0.219611 | 5.02E-13 | 1.31E-11 | 6 | 6 | 6 |
| ENSG00000214401 | KANSL1-AS1 | -5.716892 | -0.04974 | 1.08E-08 | 0.446322 | 0.588241 | 1.58E-42 | 9.23E-40 | 31 | 33 | 33 |
| ENSG00000130208 | APOC1 | 5.602462347 | 0.23838 | 2.11E-08 | 0.025396 | 0.030186 | 0.011082 | 0.054101 | 17 | 17 | 17 |
| ENSG00000103642 | LACTB | 5.48847471 | 0.139709 | 4.05E-08 | 0.051491 | 0.073098 | 6.41E-05 | 0.000539 | 20 | 20 | 20 |
| ENSG00000145217 | SLC26A1 | -5.455235824 | -0.05397 | 4.89E-08 | 0.323622 | 0.498623 | 1.80E-33 | 4.06E-31 | 13 | 13 | 13 |
| ENSG00000103510 | KAT8 | -5.392386026 | -0.11607 | 6.95E-08 | 0.072844 | 0.128222 | 7.68E-08 | 1.04E-06 | 9 | 9 | 9 |
| ENSG00000120915 | EPHX2 | 5.384310931 | 0.175084 | 7.27E-08 | 0.032479 | 0.047228 | 0.001417 | 0.008893 | 12 | 13 | 13 |
| ENSG00000176681 | LRRC37A | -5.37696919 | -0.05303 | 7.58E-08 | 0.379241 | 0.499877 | 1.38E-33 | 3.15E-31 | 54 | 55 | 55 |
| ENSG00000110079 | MS4A4A | 5.274614313 | 0.169093 | 1.33E-07 | 0.033139 | 0.032186 | 0.008684 | 0.043888 | 22 | 22 | 22 |
| ENSG00000108556 | CHRNE | 5.263692846 | 0.064604 | 1.41E-07 | 0.203786 | 0.235991 | 5.17E-14 | 1.51E-12 | 14 | 14 | 14 |
| ENSG00000238083 | LRRC37A2 | -5.251196627 | -0.05549 | 1.51E-07 | 0.300709 | 0.569209 | 1.88E-40 | 8.77E-38 | 36 | 39 | 39 |
| ENSG00000226979 | LTA | 5.246099823 | 0.237666 | 1.55E-07 | 0.017946 | 0.023147 | 0.026398 | 0.114758 | 12 | 13 | 13 |
| ENSG00000144426 | NBEAL1 | -5.160257998 | 0.081265 | 2.47E-07 | 0.115129 | 0.196116 | 1.21E-11 | 2.70E-10 | 10 | 10 | 10 |
| ENSG00000226659 | AC021028.1 | -4.995877926 | -0.13824 | 5.86E-07 | 0.04539 | 0.060843 | 0.000278 | 0.002061 | 14 | 14 | 14 |
| ENSG00000198252 | STYX | 4.915107795 | 0.155106 | 8.87E-07 | 0.038928 | 0.056539 | 0.000465 | 0.003273 | 13 | 13 | 13 |
| ENSG00000241106 | HLA-DOB | -4.800256102 | -0.05594 | 1.58E-06 | 0.258186 | 0.355786 | 6.50E-22 | 4.08E-20 | 22 | 22 | 22 |
| ENSG00000156170 | NDUFAF6 | 4.788693747 | 0.196252 | 1.68E-06 | 0.01926 | 0.021697 | 0.031646 | 0.134071 | 12 | 12 | 12 |
| ENSG00000108528 | SLC25A11 | 4.770797164 | 0.137574 | 1.83E-06 | 0.04088 | 0.051279 | 0.000872 | 0.005772 | 18 | 18 | 18 |
| ENSG00000099365 | STX1B | -4.741017034 | -0.17502 | 2.13E-06 | 0.030769 | 0.048546 | 0.00121 | 0.007734 | 4 | 4 | 4 |
| ENSG00000177051 | FBXO46 | 4.724800049 | 0.146408 | 2.30E-06 | 0.04348 | 0.06271 | 0.000222 | 0.001682 | 21 | 21 | 21 |
| ENSG00000104983 | CCDC61 | -4.689663438 | -0.17433 | 2.74E-06 | 0.033615 | 0.036898 | 0.004905 | 0.026673 | 22 | 22 | 22 |
| ENSG00000260911 | AC135050.3 | -4.651774294 | -0.1306 | 3.29E-06 | 0.052744 | 0.117325 | 2.97E-07 | 3.71E-06 | 7 | 7 | 7 |
| ENSG00000198740 | ZNF652 | -4.572383207 | -0.24937 | 4.82E-06 | 0.011551 | 0.023921 | 0.023974 | 0.105563 | 8 | 9 | 9 |
| ENSG00000177427 | MIEF2 | 4.492326727 | 0.245145 | 7.04E-06 | 0.010867 | 0.023161 | 0.026353 | 0.114585 | 3 | 3 | 3 |
| ENSG00000103356 | EARS2 | 4.466028228 | 0.111034 | 7.97E-06 | 0.054076 | 0.107869 | 9.50E-07 | 1.10E-05 | 8 | 8 | 8 |
| ENSG00000197279 | ZNF165 | -4.465009139 | -0.12495 | 8.01E-06 | 0.045967 | 0.037385 | 0.004625 | 0.025304 | 14 | 15 | 15 |
| ENSG00000143771 | CNIH4 | -4.430316685 | -0.13634 | 9.41E-06 | 0.037679 | 0.083646 | 1.80E-05 | 0.000168 | 6 | 6 | 6 |

Table S2 TWAS-identified genes associated with coronary atherosclerosis (JTI)

| gene | gene_name | zscore | effect_size | *p*value | var_g | pred_perf_r2 | pred_perf_pval | pred_perf_qval | n_snps_used | n_snps_in_cov | n_snps_in_model |
| --- | --- | --- | --- | --- | --- | --- | --- | --- | --- | --- | --- |
| ENSG00000186567 | CEACAM19 | 9.12928 | 0.11749 | 6.90E-20 | 0.220245 | 0.292057 | 1.50E-17 | 4.13E-16 | 17 | 17 | 17 |
| ENSG00000138613 | APH1B | 8.69085 | 0.178346 | 3.60E-18 | 0.067881 | 0.064171 | 0.000187 | 0.000753 | 3 | 3 | 3 |
| ENSG00000149534 | MS4A2 | -8.49824 | -0.39351 | 1.92E-17 | 0.014941 | 0.049128 | 0.001128 | 0.003698 | 8 | 8 | 8 |
| ENSG00000166924 | NYAP1 | -7.85115 | -0.29281 | 4.12E-15 | 0.024125 | 0.059216 | 0.000338 | 0.00127 | 4 | 4 | 4 |
| ENSG00000266903 | AC243964.3 | 7.828716 | 0.078385 | 4.93E-15 | 0.371911 | 0.346325 | 3.07E-21 | 1.22E-19 | 6 | 6 | 6 |
| ENSG00000030582 | GRN | -7.17178 | -0.24109 | 7.40E-13 | 0.032976 | 0.057513 | 0.000414 | 0.001516 | 6 | 6 | 6 |
| ENSG00000110079 | MS4A4A | 7.171632 | 0.59681 | 7.41E-13 | 0.004826 | 0.043653 | 0.002175 | 0.006603 | 3 | 3 | 3 |
| ENSG00000270761 | AL355353.1 | -6.86041 | -0.39373 | 6.87E-12 | 0.00971 | 0.035978 | 0.005482 | 0.014736 | 3 | 3 | 3 |
| ENSG00000158869 | FCER1G | -6.7378 | -0.19981 | 1.61E-11 | 0.038922 | 0.029705 | 0.011753 | 0.028682 | 6 | 6 | 6 |
| ENSG00000103510 | KAT8 | -6.2749 | -0.07213 | 3.50E-10 | 0.255555 | 0.173441 | 2.41E-10 | 2.83E-09 | 10 | 10 | 10 |
| ENSG00000169592 | INO80E | 6.264516 | 0.079308 | 3.74E-10 | 0.205802 | 0.183641 | 6.34E-11 | 7.98E-10 | 13 | 13 | 13 |
| ENSG00000104866 | PPP1R37 | -6.20211 | -0.41199 | 5.57E-10 | 0.009643 | 0.024248 | 0.023019 | 0.051325 | 11 | 11 | 11 |
| ENSG00000255197 | AC090559.1 | -6.18573 | -0.45683 | 6.18E-10 | 0.005966 | 0.020553 | 0.036545 | 0.076471 | 7 | 7 | 7 |
| ENSG00000149927 | DOC2A | 6.182486 | 0.595085 | 6.31E-10 | 0.003582 | 0.025807 | 0.018976 | 0.043388 | 1 | 1 | 1 |
| ENSG00000161929 | SCIMP | 6.046115 | 0.122523 | 1.48E-09 | 0.084348 | 0.155157 | 2.56E-09 | 2.61E-08 | 15 | 15 | 15 |
| ENSG00000237541 | HLA-DQA2 | -5.87258 | -0.05356 | 4.29E-09 | 0.580657 | 0.684367 | 9.65E-55 | 1.32E-51 | 13 | 13 | 13 |
| ENSG00000108379 | WNT3 | -5.8644 | -0.09375 | 4.51E-09 | 0.140058 | 0.207198 | 2.73E-12 | 4.14E-11 | 14 | 14 | 14 |
| ENSG00000158864 | NDUFS2 | -5.8561 | -0.18391 | 4.74E-09 | 0.030818 | 0.032703 | 0.008155 | 0.02084 | 8 | 8 | 8 |
| ENSG00000103642 | LACTB | 5.655853 | 0.084952 | 1.55E-08 | 0.148782 | 0.145924 | 8.30E-09 | 7.82E-08 | 9 | 10 | 10 |
| ENSG00000214401 | KANSL1-AS1 | -5.61297 | -0.04841 | 1.99E-08 | 0.437848 | 0.621416 | 2.18E-46 | 1.14E-43 | 23 | 25 | 25 |
| ENSG00000238083 | LRRC37A2 | -5.51766 | -0.05575 | 3.44E-08 | 0.323662 | 0.618062 | 5.53E-46 | 2.76E-43 | 28 | 31 | 31 |
| ENSG00000254952 | LINC02705 | 5.444775 | 0.219545 | 5.19E-08 | 0.020684 | 0.035478 | 0.005824 | 0.015506 | 3 | 3 | 3 |
| ENSG00000099381 | SETD1A | -5.32278 | -0.2437 | 1.02E-07 | 0.01876 | 0.039153 | 0.003737 | 0.010576 | 7 | 7 | 7 |
| ENSG00000108556 | CHRNE | 5.307138 | 0.05961 | 1.11E-07 | 0.245377 | 0.370576 | 5.50E-23 | 2.57E-21 | 11 | 11 | 11 |
| ENSG00000144426 | NBEAL1 | 5.260994 | 0.066096 | 1.43E-07 | 0.1889 | 0.243967 | 1.68E-14 | 3.33E-13 | 17 | 17 | 17 |
| ENSG00000234327 | AC012146.1 | -5.20033 | -0.06394 | 1.99E-07 | 0.21625 | 0.373251 | 3.49E-23 | 1.66E-21 | 12 | 12 | 12 |
| ENSG00000120915 | EPHX2 | 5.106448 | 0.07239 | 3.28E-07 | 0.176191 | 0.142456 | 1.29E-08 | 1.18E-07 | 8 | 8 | 8 |
| ENSG00000145217 | SLC26A1 | -5.10471 | -0.04376 | 3.31E-07 | 0.428164 | 0.543471 | 8.79E-38 | 1.92E-35 | 9 | 9 | 9 |
| ENSG00000139629 | GALNT6 | -5.05677 | -0.25068 | 4.26E-07 | 0.013925 | 0.052089 | 0.000792 | 0.002703 | 6 | 6 | 6 |
| ENSG00000226979 | LTA | 5.054114 | 0.221734 | 4.32E-07 | 0.019409 | 0.033625 | 0.007291 | 0.018895 | 6 | 6 | 6 |
| ENSG00000213413 | PVRIG | -5.04889 | -0.21591 | 4.44E-07 | 0.019005 | 0.022105 | 0.03007 | 0.064647 | 5 | 5 | 5 |
| ENSG00000205710 | C17orf107 | 5.015729 | 0.076849 | 5.28E-07 | 0.132368 | 0.233818 | 7.01E-14 | 1.29E-12 | 13 | 14 | 14 |
| ENSG00000156170 | NDUFAF6 | 5.000182 | 0.25935 | 5.73E-07 | 0.014491 | 0.062983 | 0.000215 | 0.000854 | 2 | 2 | 2 |
| ENSG00000090238 | YPEL3 | -4.92173 | -0.04818 | 8.58E-07 | 0.3767 | 0.27992 | 9.21E-17 | 2.34E-15 | 35 | 35 | 35 |
| ENSG00000167394 | ZNF668 | 4.756824 | 0.250592 | 1.97E-06 | 0.011652 | 0.023984 | 0.023785 | 0.052799 | 3 | 3 | 3 |
| ENSG00000073008 | PVR | -4.75131 | -0.20035 | 2.02E-06 | 0.024257 | 0.042397 | 0.002529 | 0.007526 | 1 | 1 | 1 |
| ENSG00000146094 | DOK3 | 4.747536 | 0.174783 | 2.06E-06 | 0.035699 | 0.088534 | 9.99E-06 | 5.42E-05 | 3 | 3 | 3 |
| ENSG00000260634 | AC012508.2 | 4.744629 | 0.107883 | 2.09E-06 | 0.063253 | 0.059363 | 0.000332 | 0.00125 | 9 | 10 | 10 |
| ENSG00000103356 | EARS2 | 4.721995 | 0.073145 | 2.34E-06 | 0.139273 | 0.16581 | 6.50E-10 | 7.16E-09 | 3 | 3 | 3 |
| ENSG00000260911 | AC135050.3 | -4.71838 | -0.09484 | 2.38E-06 | 0.10901 | 0.114471 | 4.22E-07 | 2.97E-06 | 4 | 4 | 4 |
| ENSG00000180787 | ZFP3 | -4.64852 | -1.06228 | 3.34E-06 | 0.000721 | 0.0349 | 0.006247 | 0.016494 | 3 | 3 | 3 |
| ENSG00000178226 | PRSS36 | 4.644119 | 0.121655 | 3.42E-06 | 0.051476 | 0.056944 | 0.000443 | 0.00161 | 3 | 3 | 3 |
| ENSG00000099817 | POLR2E | 4.637421 | 0.077108 | 3.53E-06 | 0.138718 | 0.232742 | 8.14E-14 | 1.49E-12 | 7 | 7 | 7 |
| ENSG00000198740 | ZNF652 | -4.63034 | -0.11243 | 3.65E-06 | 0.057981 | 0.062161 | 0.000237 | 0.000933 | 13 | 13 | 13 |
| ENSG00000108528 | SLC25A11 | 4.622366 | 0.141768 | 3.79E-06 | 0.036095 | 0.01857 | 0.04699 | 0.094888 | 7 | 7 | 7 |
| ENSG00000198252 | STYX | 4.602398 | 0.075265 | 4.18E-06 | 0.134453 | 0.123543 | 1.37E-07 | 1.05E-06 | 11 | 11 | 11 |
| ENSG00000177427 | MIEF2 | 4.599191 | 0.175799 | 4.24E-06 | 0.023554 | 0.060881 | 0.000277 | 0.001068 | 9 | 9 | 9 |
| ENSG00000104983 | CCDC61 | -4.56964 | -0.16773 | 4.89E-06 | 0.034353 | 0.045103 | 0.001828 | 0.005648 | 18 | 18 | 18 |

Table S3 TWAS-identified genes associated with coronary atherosclerosis (UTMOST)

| gene | gene_name | zscore | effect_size | pvalue | var_g | pred_perf_r2 | pred_perf_pval | pred_perf_qval | n_snps_used | n_snps_in_cov | n_snps_in_model |
| --- | --- | --- | --- | --- | --- | --- | --- | --- | --- | --- | --- |
| ENSG00000186567 | CEACAM19 | 9.435871429 | 0.163048 | 3.88E-21 | 0.116986 | 0.268 | 5.53E-16 | 1.58E-14 | 66 | 67 | 67 |
| ENSG00000149534 | MS4A2 | -8.800511528 | -0.67886 | 1.36E-18 | 0.005409 | 0.026 | 0.018751 | 0.061336 | 7 | 7 | 7 |
| ENSG00000166924 | NYAP1 | -7.640125065 | -0.57774 | 2.17E-14 | 0.005865 | 0.059 | 0.000339 | 0.001764 | 2 | 2 | 2 |
| ENSG00000266903 | AC243964.3 | 7.352140624 | 0.096542 | 1.95E-13 | 0.21975 | 0.336 | 1.51E-20 | 6.77E-19 | 9 | 9 | 9 |
| ENSG00000030582 | GRN | -7.008598211 | -0.2863 | 2.41E-12 | 0.022615 | 0.052 | 0.000758 | 0.003637 | 2 | 2 | 2 |
| ENSG00000169592 | INO80E | 6.59224498 | 0.10365 | 4.33E-11 | 0.138733 | 0.135 | 3.15E-08 | 3.46E-07 | 30 | 30 | 30 |
| ENSG00000178226 | PRSS36 | 6.566479163 | 0.255475 | 5.15E-11 | 0.024399 | 0.059 | 0.000361 | 0.001869 | 19 | 19 | 19 |
| ENSG00000149927 | DOC2A | 6.138153553 | 0.302624 | 8.35E-10 | 0.014056 | 0.036 | 0.005224 | 0.020141 | 14 | 14 | 14 |
| ENSG00000108379 | WNT3 | -6.134932186 | -0.13364 | 8.52E-10 | 0.074619 | 0.185 | 5.03E-11 | 7.98E-10 | 18 | 19 | 19 |
| ENSG00000103510 | KAT8 | -6.083221045 | -0.10291 | 1.18E-09 | 0.122565 | 0.147 | 7.02E-09 | 8.45E-08 | 35 | 35 | 35 |
| ENSG00000090238 | YPEL3 | -6.054915944 | -0.08099 | 1.40E-09 | 0.188256 | 0.238 | 4.02E-14 | 9.31E-13 | 14 | 14 | 14 |
| ENSG00000106261 | ZKSCAN1 | -5.895249639 | -0.46203 | 3.74E-09 | 0.006808 | 0.027 | 0.015853 | 0.053069 | 11 | 11 | 11 |
| ENSG00000234327 | AC012146.1 | -5.654133159 | -0.08598 | 1.57E-08 | 0.15005 | 0.329 | 4.82E-20 | 2.07E-18 | 89 | 90 | 90 |
| ENSG00000237541 | HLA-DQA2 | -5.475451984 | -0.05313 | 4.36E-08 | 0.446056 | 0.67 | 1.12E-52 | 1.77E-49 | 77 | 85 | 85 |
| ENSG00000156170 | NDUFAF6 | 5.430773325 | 0.274522 | 5.61E-08 | 0.013944 | 0.029 | 0.012699 | 0.043749 | 8 | 8 | 8 |
| ENSG00000214401 | KANSL1-AS1 | -5.367472902 | -0.05324 | 7.98E-08 | 0.341999 | 0.604 | 2.87E-44 | 1.78E-41 | 64 | 67 | 67 |
| ENSG00000103642 | LACTB | 5.344415758 | 0.134266 | 9.07E-08 | 0.054606 | 0.132 | 4.96E-08 | 5.30E-07 | 17 | 18 | 18 |
| ENSG00000108556 | CHRNE | 5.332672891 | 0.064017 | 9.68E-08 | 0.220642 | 0.353 | 1.04E-21 | 5.25E-20 | 36 | 37 | 37 |
| ENSG00000176681 | LRRC37A | -5.265796822 | -0.05598 | 1.40E-07 | 0.320436 | 0.459 | 5.30E-30 | 7.05E-28 | 35 | 36 | 36 |
| ENSG00000238083 | LRRC37A2 | -5.190362739 | -0.06338 | 2.10E-07 | 0.2244 | 0.593 | 4.94E-43 | 2.74E-40 | 55 | 59 | 59 |
| ENSG00000205710 | C17orf107 | 5.187442546 | 0.10242 | 2.13E-07 | 0.084826 | 0.182 | 7.83E-11 | 1.22E-09 | 95 | 96 | 96 |
| ENSG00000149922 | TBX6 | -5.090387871 | -0.06498 | 3.57E-07 | 0.210821 | 0.297 | 6.83E-18 | 2.38E-16 | 15 | 15 | 15 |
| ENSG00000174943 | KCTD13 | -5.078492101 | -0.32305 | 3.80E-07 | 0.008681 | 0.02 | 0.040892 | 0.119067 | 9 | 9 | 9 |
| ENSG00000144426 | NBEAL1 | -5.021609745 | 0.12823 | 5.12E-07 | 0.045263 | 0.205 | 3.59E-12 | 6.68E-11 | 22 | 22 | 22 |
| ENSG00000145217 | SLC26A1 | -5.002947303 | -0.05117 | 5.65E-07 | 0.303031 | 0.529 | 2.21E-36 | 5.89E-34 | 17 | 18 | 18 |
| ENSG00000130202 | NECTIN2 | -4.953805083 | -0.2282 | 7.28E-07 | 0.021831 | 0.099 | 2.75E-06 | 2.21E-05 | 19 | 19 | 19 |
| ENSG00000260625 | AC026471.2 | -4.947024341 | -0.70518 | 7.54E-07 | 0.001712 | 0.024 | 0.023092 | 0.073188 | 32 | 32 | 32 |
| ENSG00000120915 | EPHX2 | 4.862301272 | 0.100835 | 1.16E-06 | 0.084031 | 0.136 | 2.81E-08 | 3.11E-07 | 28 | 28 | 28 |
| ENSG00000161929 | SCIMP | 4.847158328 | 0.131911 | 1.25E-06 | 0.048328 | 0.088 | 1.04E-05 | 7.48E-05 | 47 | 48 | 48 |
| ENSG00000166128 | RAB8B | -4.840625685 | -0.72913 | 1.29E-06 | 0.001559 | 0.027 | 0.015577 | 0.052301 | 12 | 12 | 12 |
| ENSG00000103356 | EARS2 | 4.682707419 | 0.090109 | 2.83E-06 | 0.093168 | 0.13 | 6.40E-08 | 6.68E-07 | 10 | 10 | 10 |
| ENSG00000128923 | MINDY2 | 4.661210892 | 1.316057 | 3.14E-06 | 0.000462 | 0.121 | 1.84E-07 | 1.79E-06 | 5 | 5 | 5 |
| ENSG00000007255 | TRAPPC6A | -4.606787296 | -0.25738 | 4.09E-06 | 0.012615 | 0.063 | 0.000204 | 0.00112 | 4 | 4 | 4 |
| ENSG00000226659 | AC021028.1 | -4.604356361 | -0.10593 | 4.14E-06 | 0.061587 | 0.083 | 1.92E-05 | 0.000131 | 22 | 22 | 22 |
| ENSG00000260136 | AC008915.2 | -4.589736776 | -0.18518 | 4.44E-06 | 0.022893 | 0.043 | 0.002264 | 0.009673 | 29 | 30 | 30 |
| ENSG00000265148 | TSPOAP1-AS1 | -4.567515061 | -0.57365 | 4.94E-06 | 0.002086 | 0.03 | 0.011244 | 0.039438 | 11 | 13 | 13 |
| ENSG00000151006 | PRSS53 | 4.528720365 | 0.151089 | 5.93E-06 | 0.031689 | 0.042 | 0.00255 | 0.010733 | 20 | 20 | 20 |
| ENSG00000260911 | AC135050.3 | -4.507893308 | -0.10477 | 6.55E-06 | 0.072062 | 0.09 | 8.53E-06 | 6.27E-05 | 22 | 22 | 22 |

Table S4 TWAS-identified genes associated with coronary atherosclerosis (FUSION)

| ID | CHR | P0 | P1 | HSQ | BEST.GWAS.ID | BEST.GWAS.Z | EQTL.ID | EQTL.R2 | EQTL.Z | EQTL.GWAS.Z | NSNP | NWGT | MODEL | MODELCV.R2 | MODELCV.PV | TWAS.Z | TWAS.P |  |
| --- | --- | --- | --- | --- | --- | --- | --- | --- | --- | --- | --- | --- | --- | --- | --- | --- | --- | --- |
| ENSG00000112137.17 | 6 | 12716804 | 12716805 | 0.2271 | rs9349379 | 13.2 | rs9349379 | 1.04E-01 | -4.96 | 13.201 | 493 | 1 | top1 | 0.1 | 7.90E-06 | -13.201 | 8.66E-40 | PHACTR1 |
| ENSG00000144426.18 | 2 | 2.03E+08 | 2.03E+08 | 0.3961 | rs6705330 | 7.651 | rs4675310 | 0.258376 | -7.06 | 7.625 | 261 | 261 | susie | 0.27 | 1.30E-13 | -7.62538 | 2.43E-14 | NBEAL1 |
| ENSG00000153774.8 | 16 | 75433484 | 75433485 | 0.2312 | rs2161684 | -7.27 | rs8046416 | 0.099091 | 4.69 | -6.905 | 347 | 1 | top1 | 0.099 | 1.30E-05 | -6.905 | 5.02E-12 | CFDP1 |
| ENSG00000168906.12 | 2 | 85539164 | 85539165 | 0.3333 | rs17026396 | 7.748 | rs699664 | 0.093813 | 4.62 | 5.615 | 480 | 480 | susie | 0.12 | 1.30E-06 | 6.65643 | 2.81E-11 | MAT2A |
| ENSG00000164694.16 | 6 | 1.59E+08 | 1.59E+08 | 0.1531 | rs905684 | 6.56 | rs7744848 | 4.26E-02 | 4.35 | 6.402 | 566 | 3 | lasso | 0.076 | 0.00013 | 6.3988 | 1.57E-10 | FNDC1 |
| ENSG00000264801.1 | 9 | 21929456 | 21929457 | 0.1675 | rs1333042 | 23.4 | rs7023329 | -5.76E-03 | -4.02 | -6.081 | 437 | 437 | susie | 0.006 | 0.15 | 6.27518 | 3.49E-10 | ERVFRD-3 |
| ENSG00000147889.17 | 9 | 21995300 | 21995301 | 0.1611 | rs1333042 | 23.4 | rs16938590 | -6.85E-05 | 3.28 | 2.301 | 447 | 447 | susie | 0.036 | 0.0066 | 6.22786 | 4.73E-10 | CDKN2A |
| ENSG00000272750.1 | 1 | 2.23E+08 | 2.23E+08 | 0.2008 | rs2291832 | -6.17 | rs2133189 | 0.160674 | 5.53 | -6.137 | 328 | 1 | top1 | 0.16 | 2.30E-08 | -6.137 | 8.41E-10 |  |
| ENSG00000261783.1 | 16 | 75381259 | 75381260 | 0.4612 | rs2161684 | -7.27 | rs166013 | 0.405541 | 8.45 | -5.908 | 354 | 1 | top1 | 0.41 | 1.70E-21 | -5.908 | 3.46E-09 |  |
| ENSG00000183520.11 | 1 | 38009257 | 38009258 | 0.183 | rs11485595 | 6.36 | rs17531077 | 0.10854 | 5.03 | -5.857 | 464 | 1 | top1 | 0.11 | 5.10E-06 | -5.857 | 4.71E-09 | UTP11 |
| ENSG00000183386.9 | 1 | 38005605 | 38005606 | 0.2706 | rs11485595 | 6.36 | rs28435150 | 0.198357 | 6.1 | -5.759 | 466 | 466 | susie | 0.2 | 2.10E-10 | -5.69711 | 1.22E-08 | FHL3 |
| ENSG00000136378.14 | 15 | 78811430 | 78811431 | 0.212 | rs12906835 | 9.69 | rs11072810 | 0.071326 | 4.33 | -5.21 | 416 | 416 | susie | 0.074 | 0.00015 | -5.61112 | 2.01E-08 | ADAMTS7 |
| ENSG00000158186.12 | 3 | 1.38E+08 | 1.38E+08 | 0.381 | rs6807945 | 6.86 | rs9828345 | 0.159548 | 6 | 3.256 | 305 | 8 | enet | 0.18 | 4.10E-09 | 5.5656 | 2.61E-08 | MRAS |
| ENSG00000283050.1 | 4 | 1.19E+08 | 1.19E+08 | 0.228 | rs13139045 | -6.12 | rs10518329 | 0.121922 | 5.69 | -5.564 | 414 | 1 | top1 | 0.12 | 1.30E-06 | -5.564 | 2.64E-08 | GTF2IP12 |
| ENSG00000183431.11 | 1 | 37990920 | 37990921 | 0.3318 | rs11485595 | 6.36 | rs7366048 | 0.200903 | 6.13 | -5.797 | 471 | 6 | lasso | 0.2 | 2.90E-10 | -5.25019 | 1.52E-07 | SF3A3 |
| ENSG00000166848.5 | 16 | 75647785 | 75647786 | 0.1566 | rs2161684 | -7.27 | rs11641943 | 0.020299 | -3.04 | 5.039 | 400 | 1 | top1 | 0.02 | 0.033 | -5.039 | 4.68E-07 | TERF2IP |
| ENSG00000118526.6 | 6 | 1.34E+08 | 1.34E+08 | 0.2136 | rs1209415 | 6.75 | rs12193973 | 6.30E-02 | 5.14 | -5.145 | 430 | 430 | susie | 0.087 | 4.20E-05 | -4.9856 | 6.18E-07 | TCF21 |
| ENSG00000174953.13 | 3 | 1.54E+08 | 1.54E+08 | 0.156 | rs1357079 | -5.28 | rs355780 | 0.041466 | 4.57 | -4.877 | 325 | 325 | susie | 0.082 | 7.10E-05 | -4.9569 | 7.16E-07 | DHX36 |
| ENSG00000158604.14 | 7 | 44582286 | 44582287 | 0.1583 | rs732797 | -4.91 | rs732797 | 0.05611 | -3.89 | -4.906 | 364 | 1 | top1 | 0.056 | 0.00093 | 4.906 | 9.30E-07 | TMED4 |
| ENSG00000245958.6 | 4 | 1.19E+08 | 1.19E+08 | 0.2821 | rs13139045 | -6.12 | rs6846442 | 0.274261 | -7.47 | -4.699 | 414 | 414 | susie | 0.31 | 6.80E-16 | 4.7967 | 1.61E-06 | SEPTIN7P14 |
| ENSG00000102967.11 | 16 | 72008587 | 72008588 | 0.3405 | rs11864453 | 5.98 | rs7195958 | 0.171527 | -5.56 | -4.73 | 369 | 1 | top1 | 0.17 | 7.30E-09 | 4.73 | 2.25E-06 | DHODH |
| ENSG00000263335.1 | 16 | 15726673 | 15726674 | 0.27 | rs10521101 | 5.02 | rs16967494 | 0.258569 | 6.77 | 4.613 | 458 | 1 | top1 | 0.26 | 4.10E-13 | 4.613 | 3.97E-06 |  |
| ENSG00000105889.14 | 7 | 22632924 | 22632925 | 0.2074 | rs6969258 | 4.5 | rs6969258 | 0.121788 | 5.32 | 4.499 | 545 | 545 | susie | 0.13 | 3.80E-07 | 4.5036 | 6.68E-06 | STEAP1B |
| ENSG00000179428.2 | 7 | 22726704 | 22726705 | 0.4973 | rs6969258 | 4.5 | rs2069832 | 0.391623 | 8.61 | 4.34 | 514 | 514 | susie | 0.42 | 1.70E-22 | 4.4384 | 9.06E-06 | IL6-AS1 |
| ENSG00000224609.6 | 1 | 59208120 | 59208121 | 0.4258 | rs12758643 | -4.39 | rs12752853 | 0.315808 | 7.53 | -4.388 | 400 | 1 | top1 | 0.32 | 3.60E-16 | -4.388 | 1.14E-05 | FGGY-DT |

Table S5 causal relationships between *NBEAL1* gene and coronary atherosclerosis

| probeID | ProbeChr | Gene | Probe_bp | topSNP | A1 | A2 | Freq | b_GWAS | se_GWAS | p_GWAS | b_eQTL | se_eQTL | p_eQTL | b_SMR | se_SMR | p_SMR | p_HEIDI | nsnp_HEIDI |
| --- | --- | --- | --- | --- | --- | --- | --- | --- | --- | --- | --- | --- | --- | --- | --- | --- | --- | --- |
| ENSG00000144426 | 2 | NBEAL1 | 203879602 | rs140201293 | C | T | 0.12 | 0.0998 | 0.013 | 6.19E-14 | -0.578 | 0.073 | 4.23E-15 | -0.172 | 0.032 | 5.85E-08 | 0.611 | 13 |
