# Supplementary figures and images for: Transcriptome-wide association study reveals novel susceptibility genes for coronary atherosclerosis
Source: Front Cardiovasc Med. 2023 Jun 7;10:1149113. doi: 10.3389/fcvm.2023.1149113 (PMC10282549; doi:10.3389/fcvm.2023.1149113)

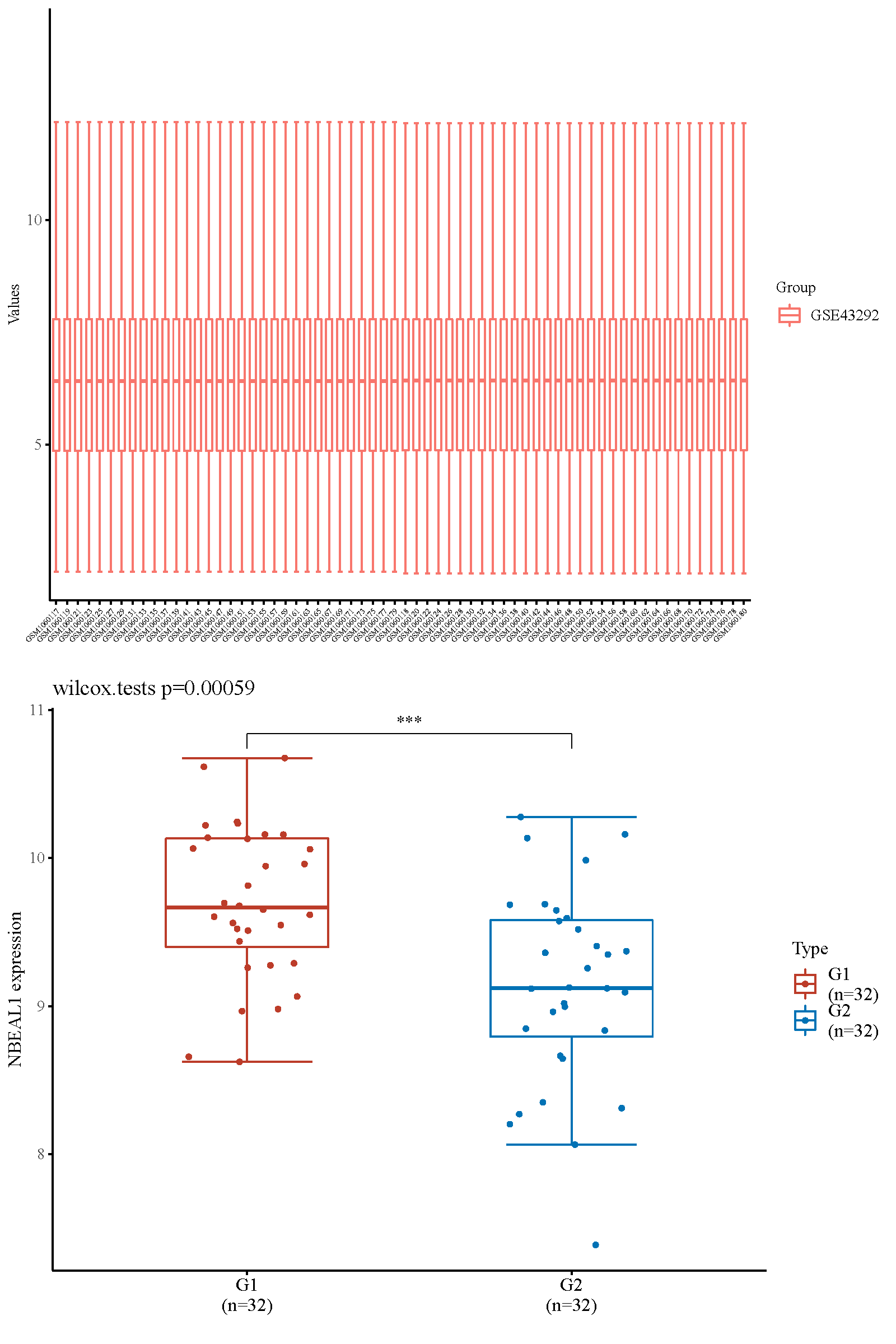

Supplement: Supplementary file 2 [file Image1.tif]
